# Supplementary figures and images for: IL-22BP production is heterogeneously distributed in Crohn’s disease
Source: Front Immunol. 2022 Oct 13;13:1034570. doi: 10.3389/fimmu.2022.1034570 (PMC9612839; doi:10.3389/fimmu.2022.1034570)

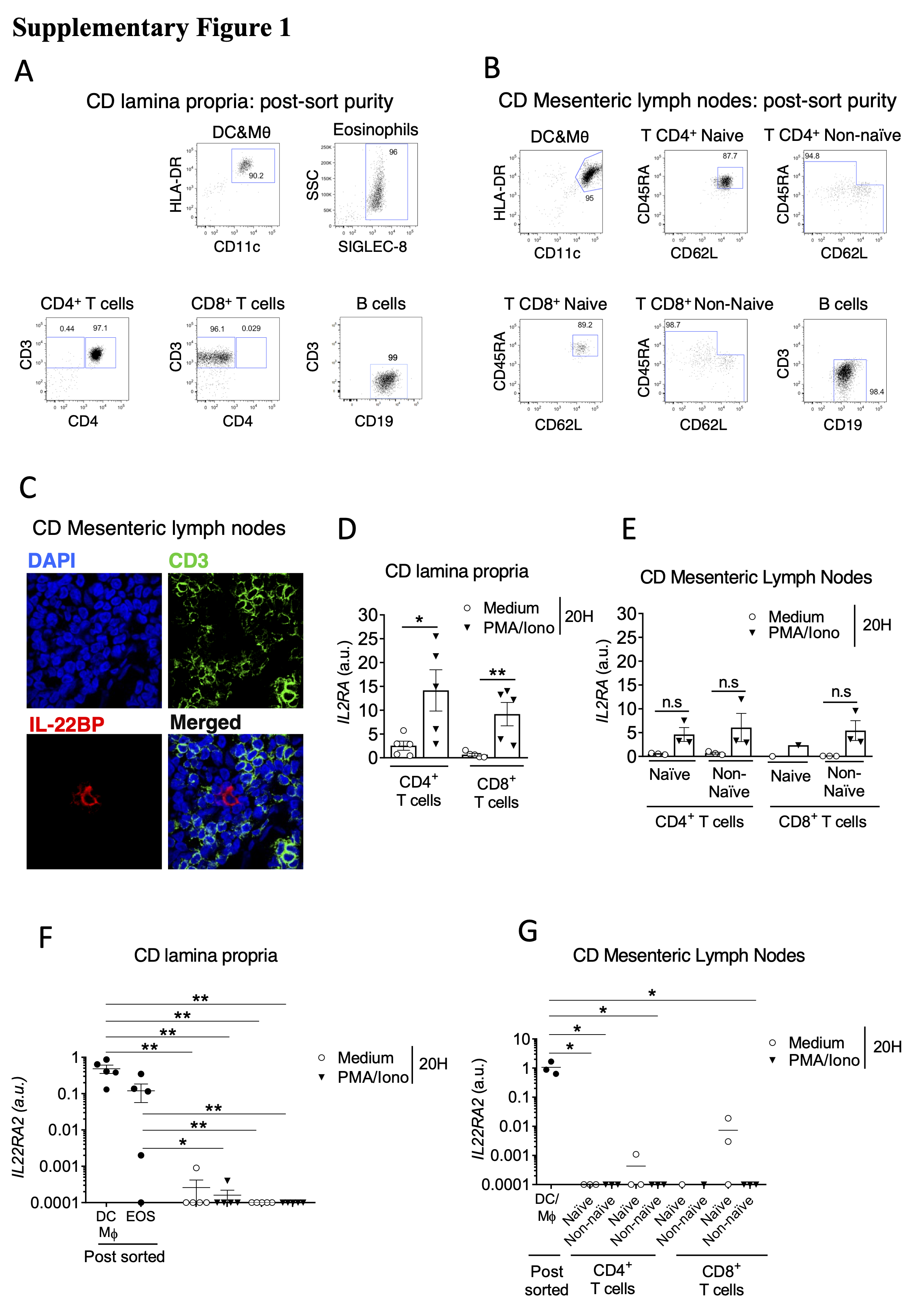

Supplement: Supplementary Figure 1 — (A, B) Representative dot plots showing the post-sort purity of indicated cell subsets sorted from the lamina propria of intestinal surgical resections (A) and mesenteric lymph nodes (MLN) (B) of CD patients. (C) Representative IFI pictures of slides from formalin-fixed, paraffin-embedded (FFPE) sections of CD MLN stained with mAbs against IL-22BP (red) and CD3 (green), as well as with DAPI (blue) (n=3 CD patients). Original magnification x 630. (D–G) Indicated T cell subsets were sorted from CD lamina propria intestinal surgical resections (n=5) (D, F) and MLN (n=3) (E, G) and cultured with or without PMA (50ng/mL) and Ionomycin (250ng/mL) (PMA/Iono). After 20 hours, expressions of IL2RA (D–E) and IL22RA2 (F–G) were analyzed by RT-qPCR. IL22RA2 expression levels of freshly isolated DC/Macrophages and eosinophils are indicated for reference. Means comparisons were performed with the Kruskal–Wallis test for unpaired samples and with the Wilcoxon test for paired samples. P-value < 0.05 were considered statistically significant. CD: Crohn’s disease; DC: dendritic cells, EOS: eosinophils; Mθ: macrophages. [file Image_1.tiff]
